# Integrating ATAC-Seq and RNA-Seq Reveals the Signal Regulation Involved in the *Artemia* Embryonic Reactivation Process

**DOI:** 10.3390/genes15081083

**Published:** 2024-08-16

**Authors:** Anqi Li, Zhentao Song, Mingzhi Zhang, Hu Duan, Liying Sui, Bin Wang, Tong Hao

**Affiliations:** 1 Tianjin Key Lab of Aqua-Ecology and Aquaculture, Fisheries College, Tianjin Agricultural University, Tianjin 300384, China; anqi826523329@163.com; 2 Tianjin Key Laboratory of Animal and Plant Resistance, College of Life Sciences, Tianjin Normal University, Tianjin 300387, China; a953835912@gmail.com (Z.S.); 18404964001@163.com (M.Z.); wbkjc@tjnu.edu.cn (B.W.); 3 College of Marine and Environmental Sciences, Tianjin University of Science & Technology, Tianjin 300222, China; duanhxh@tust.edu.cn (H.D.); suily@tust.edu.cn (L.S.)

**Keywords:** embryonic reactivation, embryonic diapause, signal transduction, G-protein-coupled receptor, *Artemia* cyst

## Abstract

Embryonic diapause is a common evolutionary adaptation observed across a wide range of organisms. *Artemia* is one of the classic animal models for diapause research. The current studies of *Artemia* diapause mainly focus on the induction and maintenance of the embryonic diapause, with little research on the molecular regulatory mechanism of *Artemia* embryonic reactivation. The first 5 h after embryonic diapause breaking has been proved to be most important for embryonic reactivation in *Artemia*. In this work, two high-throughput sequencing methods, ATAC-seq and RNA-seq, were integrated to study the signal regulation process in embryonic reactivation of *Artemia* at 5 h after diapause breaking. Through the GO and KEGG enrichment analysis of the high-throughput datasets, it was showed that after 5 h of diapause breaking, the metabolism and regulation of *Artemia* cyst were quite active. Several signal transduction pathways were identified in the embryonic reactivation process, such as G-protein-coupled receptor (GPCR) signaling pathway, cell surface receptor signaling pathway, hormone-mediated signaling pathway, Wnt, Notch, mTOR signaling pathways, etc. It indicates that embryonic reactivation is a complex process regulated by multiple signaling pathways. With the further protein structure analysis and RT-qPCR verification, 11 GPCR genes were identified, in which 5 genes function in the embryonic reactivation stage and the other 6 genes contribute to the diapause stage. The results of this work reveal the signal transduction pathways and GPCRs involved in the embryonic reactivation process of *Artemia* cysts. These findings offer significant clues for in-depth research on the signal regulatory mechanisms of the embryonic reactivation process and valuable insights into the mechanism of animal embryonic diapause.

## 1. Introduction

Embryonic diapause is a widespread biological phenomenon, which has been reported from plants to mammals [1,2]. It is also one of the most common forms of diapause in Arthropods. Embryonic diapause is marked by decreased metabolism, the accumulation of nutrient reserves, halted development, reduced water content, and enhanced resilience to environmental and physiological stress [3]. The entire diapause process can be segmented into three stages: pre-diapause, diapause, and post-diapause stages [4]. The onset of diapause stages is typically initiated by specific environmental cues, including changes in photoperiod, temperature, food scarcity, or crowding. Additionally, some animals enter diapause as a compulsory aspect of their lifecycle, independent of external triggers. The diapause situation maintains until the appearance of an environmental stimuli and then enters the post-diapause stage [5]. In the post-diapause stage, the embryo can be activated in a short period of time and quickly begins the process of continuing development triggered by the appropriate environmental signal stimulation.

**Figure 1:**
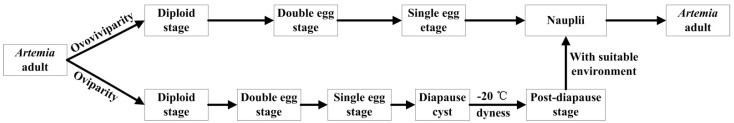
The lifespan of *Artemia*.

*Artemia*, also named as brine shrimp, is a small planktonic crustacean animal and attractive living food for fishes and crustaceans, which is one of the classic animal models for diapause research [6]. *Artemia* exhibit two reproductive modes within a single individual’s lifespan: oviparity, which produces resting eggs (cysts), and ovoviviparity, which results in free-swimming nauplii (Figure 1). Females have the capability to switch between these two reproductive modes [7]. Under the conditions of suitable temperature, light, oxygen, and sufficient food, *Artemia* goes through the reproductive mode of ovoviviparity. Firstly, the oocytes located on both sides of the reproductive nodes gradually develop and enlarge over 2–3 days, entering the diploid stage. Then, the oocytes on both sides move into the fallopian tubes over 3–4 days, forming two white cell clusters called the double egg stage. The double egg stage oocytes enter the uterus and form an embryo, known as the single egg stage. After nearly a week of development, they directly give birth to free-swimming nauplii. When environmental conditions become harsh, such as low temperature, hypoxia, and lack of food, *Artemia* reproduce by oviparity. Similar to the process of ovoviviparity, oviparous *Artemia* also undergo the diploid stage, double egg stage, and single egg stage. However, unlike in ovoviviparity, after the single egg stage, they lay diapause eggs encased in a hard shell, known as cysts. Cysts have a strong protective effect against stress, which enables *Artemia* to resist harsh environmental conditions for years. Under the stimulation of −20 °C and dryness, the embryo enters post-diapause stage, which is a state of quiescence. When the environment changes to be suitable, the embryonic development is restarted and the nauplius is ultimately hatched to continue reproduction. Once the diapause is broken, it cannot be restored to the diapause state again. After the beginning of embryonic reactivation, the embryo will be hatched into a nauplius within 24 h [8]. As *Artemia* is an attractive living food and its cysts are facilitated to be stored and circulated, it has great economic value and has been commercially harvested and traded around the world. The research on the diapause cysts of *Artemia* not only provides important insights into the mechanism of animal embryonic diapause, but also plays an important role in artificially controlling the breeding of *Artemia* and improving its economic value.

The *Artemia* cyst in diapause exhibits extremely low metabolic activity. It was reported that the consumption of commonly used energy storage substances are not detected in *Artemia* during dormancy [9], but a high-energy substance named Diguanosine (Gp4G) is constantly being consumed [10]. It indicates that there are indeed traces of life activity during the hibernation process of *Artemia* cysts. Gp4G is consumed in large quantities in the early stages of embryonic activation, and it is speculated that the energy substances such as ATP and GTP can be produced from Gp4G for the early embryonic activation [10].

An important feature of *Artemia* cyst is that it has greatly enhanced stress tolerance, which is dependent on the cyst wall, trehalose, and late embryonic abundant proteins [6]. Over 20 oviparous specially expressed genes [7,11], some enzymes and proteins were found related with the formation of *Artemia* cyst and its resistance to environmental stress, such as the deubiquitinating enzyme BAP1 [12], Ste20-like kinase [13], CT10 regulator of kinase (Crk) [14], and some chitin-binding proteins [15]. In addition, the transcription cofactor p8 [16] and some diapause-specific molecular chaperones were found related with the diapause process of *Artemia*, including the small heat shock proteins (sHsps) p26 [17], ArHsp21 [18], ArHsp22 [19,20], ArHsp40-2 [21], and the ferritin homolog artemin [22]. The synthesis of the molecular chaperones is closely related to the quantity and location of a transcription factor, heat shock factor 1 (Hsf1) [23,24].

The current studies of *Artemia* diapause mainly focus on the induction and maintenance of the embryonic diapause, but there is little research on the molecular regulatory mechanism of embryonic reactivation after diapause breaking. At the molecular level, it was shown that the first 5 h after diapause breaking are the most important for embryonic reactivation based on the analysis of the ESTs and proteomic studies of *Artemia* embryos before and after diapause breaking [25]. A significant increase in differentially expressed genes (DEGs) were found during this period, and the differential expression of some important markers related to diapause were mostly concentrated in this period [26]. In addition, a nuclear factor protein DEK was found related to cyst reactivation of *Artemia* via the increase in euchromatin and decrease in heterochromatin [27]. However, the molecular mechanism of *Artemia* embryonic reactivation is still unclear.

To explore the molecular regulatory mechanism of embryonic reactivation in *Artemia*, in this work, the gene expression of *Artemia* cyst in diapause stage and 5 h after diapause breaking were tested by ATAC-seq and RNA-seq. The DEGs in the two stages were analyzed by combining the two high throughput sequencing results, especially the DEGs related to signaling transduction. The mechanism of signal regulation involved in *Artemia* embryonic reactivation was analyzed at the molecular level.

## 2. Methods

### 2.1. Identification and Analysis of DEGs

Data of ATAC-seq and RNA-seq for diapause and embryonic reactivation stages were obtained from GEO database with the accession numbers GSE248452 (ATAC-seq data) and GSE249417 (RNA-seq data). Data for the diapause stage are marked as ArD_0h, which is sampled from diapause *Artemia* cysts. Data for the embryonic reactivation stage are marked as ArR_5h, which is sampled from the reactivated cysts at 5 h after diapause breaking.

Peaks of different stages from ATAC-seq were merged using BEDTools (Charlottesville, VA, USA) [28]. The mean reads per million mapped reads (RPMs) of each group in the merge peak were calculated. Peaks with |log2.FoldEnrich| > 1 and *p*-value ≤ 0.05 were assigned as differential peaks. The genes associated with differential peaks were considered as DEGs.

Differential analysis of the genes from RNA-seq was performed with the DESeq2 R (version 1.20.0, Boston, MA, USA) [29]. The resulting *p*-values were adjusted using the Benjamini and Hochberg’s method to control the false discovery rate. While in RNA-seq results, genes with Padj ≤ 0.05 and |log2.Fold_change| > 1 were considered as DEGs.

The Gene Ontology (GO) and Kyoto Encyclopedia of Genes and Genomes (KEGG) enrichment for the DEGs were analyzed with Goseq (version 4.10.2, Parkville, Australia) [30] and KOBAS software (version 3.0, Beijing, China) [31].

### 2.2. Integration Analysis of ATAC-Seq and RNA-Seq

The DEGs in the ATAC-seq and RNA-seq datasets were compared. As the genes from the ATAC-seq may associated to multi peaks, if the peaks related shows different expression profiles, for example, one peak is up-regulated and another one is down-regulated, the expression profile in line with the same gene in RNA-seq dataset was reserved. The common genes with consistent expression profiles in both ATAC-seq and RNA-seq datasets were subjected to GO and KEGG enrichment analysis.

### 2.3. Structure Analysis of Candidate GPCR Proteins

For the purpose of further identifying the possible GPCR genes, TMHMM [32] (https://services.healthtech.dtu.dk/service.php?TMHMM-2.0, accessed on 4 February 2024) was employed to identify the trans-membrane helix (TMH) structure of the proteins corresponding to these genes. Furthermore, the PredictProtein (https://predictprotein.org/, accessed on 4 February 2024) [33] was used to predict the secondary structure of the proteins, and SWISS-MODEL (https://swissmodel.expasy.org/, accessed on 4 February 2024) [34] was utilized to predict the tertiary structures of the proteins.

### 2.4. Artemia Hatching and Culture

Artemia parthenogenetica were farmed in 30‰ artificial seawater with a photoperiod of 16 h of light and 8 h of dark per day. The diapause cytsts were treated with −20 °C and dehydration to promote them into the post-diapause stage. In order to initiate embryo activation and facilitate its continuous development, the dry cysts were thoroughly rehydrated by hatching in 30‰ artificial seawater (Blue Starfish, Hangzhou, Zhejiang, China) at 28 °C under continuous illumination, which means the beginning of embryonic reactivation of Artemia. For diapause stage and 5 h after reactivation, three distinct cyst samples for each stage were quickly collected and put in liquid nitrogen immediately and then preserved in a −80 °C refrigerator for the following RT-qPCR experiment. The samples of diapause stage were named as ArD_0h (ArD_0h_1, ArD_0h_2, ArD_0h_3), and those of 5 h after reactivation were named as ArR_5h (ArR_5h_1, ArR_5h_2, ArR_5h_3).

### 2.5. Verification of Changes in Genes Expression for Candidate GPCRs

The expressions of candidate GPCRs were validated using RT-qPCR. An 80 mg sample with individual cysts grouped together was used for each RT-qPCR experiment. All of the RT-qPCR experiments were performed in three biological replicates. RNA was extracted with TRIzol reagent (Invitrogen, San Diego, CA, USA). Samples were placed in the TRIzol reagent for lysis. Add chloroform, shake vigorously, and centrifuge. Then transfer the upper aqueous phase to a new tube, add an equal volume of isopropanol, and centrifuge again. The RNA will precipitate. Wash the RNA pellet twice with 75% ethanol, centrifuging each time and discarding the supernatant. Finally, dissolve the RNA pellet in RNase-free water and measure the RNA concentration. The reverse transcription was performed using the PrimeScriptTM RT Master Mix (Perfect Real Time) kit (Takara, Kusatsu, Japan). The reaction mixture (10 μL) comprised 2 μL 5× PrimeScript Buffer (for real time), 0.5 μL PrimeScript RT Enzyme Mix I, 0.5 μL Oligo dT Primer (50 μM), 0.5 μL random 6 mers (100 μM), total RNA less than 500 ng, and RNase Free dH2O up to 10 μL. The reaction conditions were set as follows: reverse transcription reaction at 37 °C for 15 min, followed by termination at 85 °C for 5 s and 4 °C for preservation. Fluorescence quantitative detection was conducted using the TB Green^®^ Premix Ex TaqTM II (Tli RNaseH Plus) × 2 kit (Takara, Kusatsu, Japan) on an ABI Applied Biosystems instrument. The housekeeping gene utilized was tubulin. The reaction mixture (20 μL) comprised 1 μL cDNA, 0.4 μL of each primer (10 μM), 10 μL TB Green^®^ Premix Ex TaqTM II (2×), 0.4 μL ROX Reference Dye (50×), and ddH2O up to 20 μL. The reaction conditions were set as follows: initial denaturation at 95 °C for 30 s, followed by 40 cycles of 5 s at 95 °C, 30 s at 60 °C, and 60 °C for 1 min. Primers sequences used for RT-qPCR were listed in Table S1. The data were quantitatively analyzed with 2^−ΔΔCt^ method.

## 3. Results

### 3.1. DEG Analysis of Genes from ATAC-Seq

**Figure 2:**
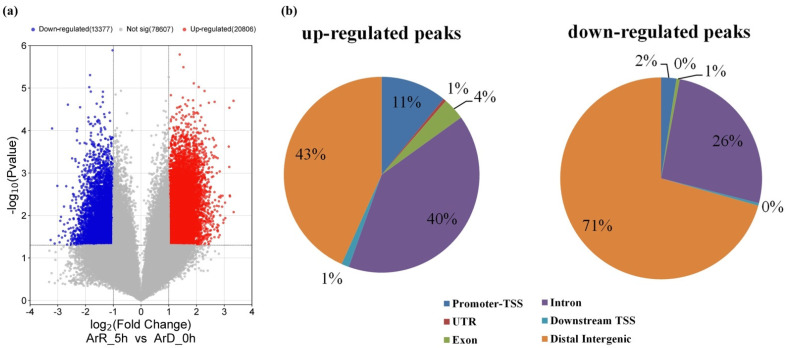
The volcano plot and genome-wide distribution of differential enriched peaks from ATAC-seq. (**a**) The volcano plot of differential enriched peaks. (**b**) The genome-wide distribution of differential enriched peaks.

The “FoldEnrich” values of each peak in the ArD_0h and ArR_5h groups were compared. In total, 34,183 differential enriched peaks were identified, in which 20,806 were up-regulated and 13,377 were down-regulated (Figure 2a). The functional regions of differential enriched peaks on the genome were annotated (Figure 2b). The TSS is normally found in the promoter-TSS region. The intron and distal intergenic regions comprised the largest portion. The promoter-TSS region was only 2% and 11% for the down-regulated and up-regulated peaks, respectively.

**Figure 3:**
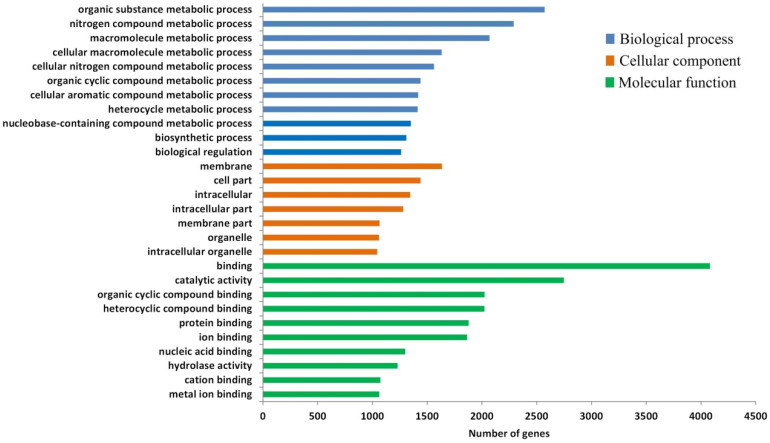
GO enrichment of DEGs from ATAC-seq.

The 34,183 differential enriched peaks between ArD_0h and ArR_5h groups associated with 9159 DEGs, in which 7124 were up-regulated and 5339 were down-regulated. The GO enrichment analysis results for the DEGs indicated that, in terms of biological process annotations, these genes were primarily enriched in biological regulation and metabolic processes, including organic substance, nitrogen compound, and macromolecule metabolic processes. Regarding cellular component annotations, the DEGs were predominantly located in the membrane and cell part. For molecular function annotations, the DEGs were largely concentrated in binding and catalytic activity (Figure 3). These results revealed that at 5 h after diapause breaking, metabolism and signal regulation processes are significantly changed in the *Artemia* cyst, with the most significant changes occurring on the cell membrane.

### 3.2. DEG Analysis of Genes from RNA-Seq

**Figure 4:**
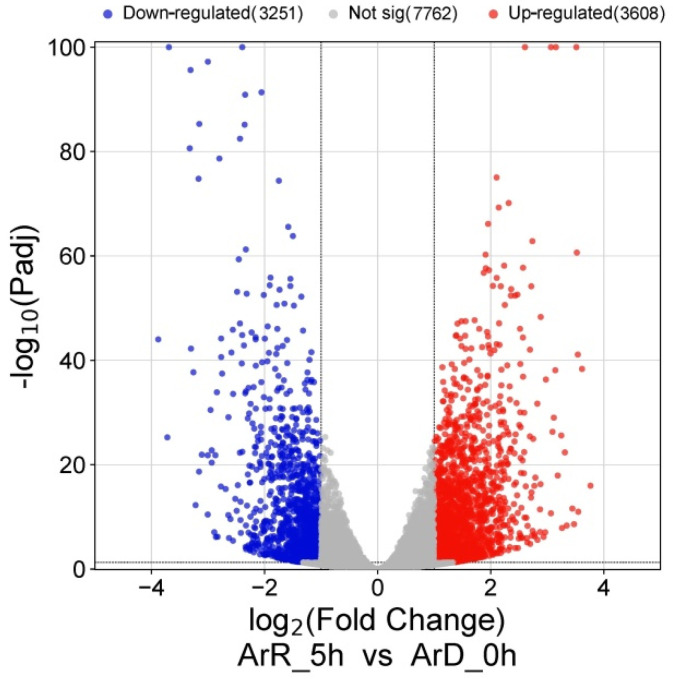
The volcano plot of DEGs from RNA-seq.

**Figure 5:**
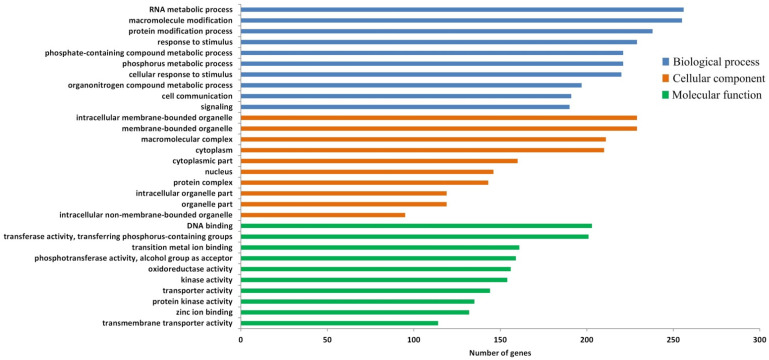
GO enrichment of DEGs from RNA-seq.

Through the DEG analysis of RNA-seq dataset, a total of 6859 DEGs were found between the ArD_0h and ArR_5h groups, in which 3608 DEGs were up-regulated and 3251 DEGs were down-regulated (Figure 4). The GO enrichment analysis of these DEGs showed that they were largely enriched in several metabolic processes, the protein modification process, response to stimulus, cell communication, and the signaling process. The cellular locations of the DEGs were mainly distributed in the membrane-bound organelle, macromolecular complex, and cytoplasm. The DEGs usually have DNA binding and transferase activity (Figure 5). These results suggested that the metabolic process undergoes significant changes in the embryonic reactivation process, and the communication between cells are active, which may be mediated by the signaling system.

### 3.3. Integration Analysis of DEGs from ATAC-Seq and RNA-Seq

**Figure 6:**
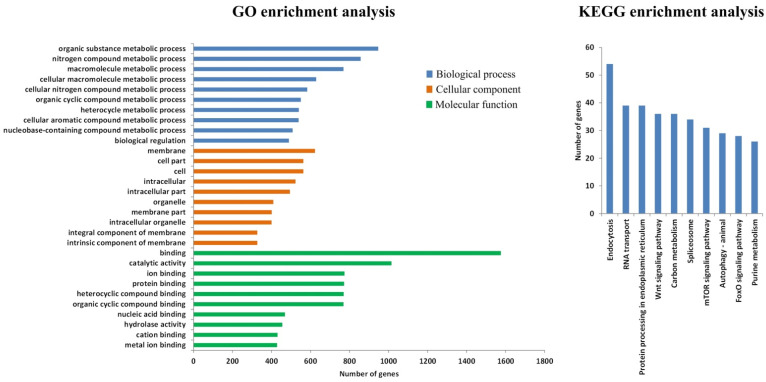
GO and KEGG enrichment analysis of integrated DEGs.

The DEGs and their expression profiles from ATAC-seq and RNA-seq datasets were compared. A total of 4137 common genes were identified, with 3116 displaying the same expression profile. These genes were subsequently referred to as integrated DEGs in the following analysis (Table 1). The remaining 1021 genes, while common, displayed inconsistent expression profiles. Of these genes, 718 were up-regulated in ATAC-seq but down-regulated in RNA-seq, and 303 were down-regulated in ATAC-seq but up-regulated in RNA-seq. The GO enrichment analysis of the integrated DEGs revealed that for the biological process annotations, these genes were largely enriched in the biological regulation and metabolic process including “organic substance”, “nitrogen compound”, “macromolecule metabolic process” etc. It indicates significant changes in *Artemia* after 5 h of reactivation. These DEGs were mostly positioned in the membrane and cell part and largely concentrated in the binding and catalytic activity (Table S2). The metabolic changes in *Artemia* cells are closely related to cellular regulation. The GO enrichment analysis indicates that the regulatory processes may be closely related to cellular membrane and proteins binding on membrane. In KEGG enrichment analysis of the DEGs, there are also many genes distributed in regulatory pathways, with Wnt, mTOR, and FoxO signaling pathways enriching the most DEGs (Figure 6, Table S2). In addition, AGE-RAGE, TGF-β, Notch signaling pathway and Phosphatidylinositol signaling system also participated in the embryonic reactivation process in *Artemia*. A total of 96 genes are involved in the signal transduction pathways, with 73 DEGs being up-regulated and 23 genes being down-regulated.

**Figure 7:**
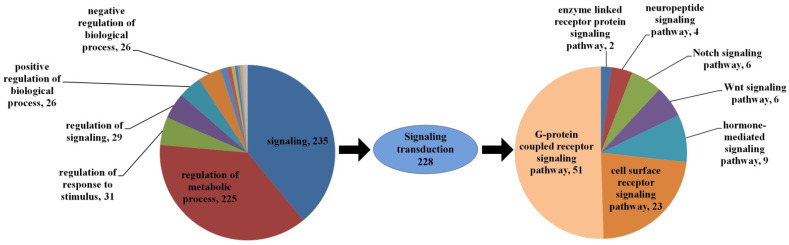
Enrichment of DEGs in the childhood of “regulation of biological process”.

For the purpose of investigating the main pathways that function in the regulation of embryonic reactivation, the distribution of DEGs in the childhood of “regulation of biological process” (GO:0050789) was further analyzed. The 452 DEGs in “regulation of biological process” are mainly distributed in “signaling” (GO:0023052, containing 235 DEGs) and “regulation of metabolic process” (GO:0019222, containing 225 DEGs) (Figure 6). In the children of signaling annotation, DEGs with signaling annotation majorly enriched in “signal transduction” (GO:0007165, containing 228 DEGs), which indicates that signal transduction plays an important role in the embryonic reactivation of *Artemia*. In the further analysis of the signal transduction DEGs, they were found to primarily function in the “G-protein-coupled receptor signaling pathway” (GO:0007186, containing 51 DEGs) and “cell surface receptor signaling pathway” (GO:0007166, containing 23 DEGs). It can be inferred that *Artemia* mainly transfer signals via certain signaling pathways when receiving signals by specific cell surface receptors, such as GPCRs. In addition, the hormone-mediated signaling pathway, Wnt, Notch, neuropeptide, and enzyme linked receptor protein signaling pathways were also involved in the regulation of embryonic reactivation (Figure 7).

In addition to the GO enrichment analysis, the KEGG pathway enrichment was also analyzed for the 235 DEGs enriched in “signaling” (GO:0023052). It was found that 16 DEGs were located in signaling pathways distributed in mTOR (6 DEGs), AGE-RAGE (4 DEGs), FoxO (3 DEGs), Notch (1 DEG), and Phosphatidylinositol signaling system (2 DEGs). Although the genes with KEGG annotations were far fewer than those with GO annotations, the KEGG enrichment analysis to some extent provide Supplementary Information for the investigation of the active pathways in embryonic reactivation.

Among the 51 DEGs in the G-protein-coupled receptor signaling pathway, 32 were found to possess “GPCR activity” (GO:0004930), and 30 genes had “transmembrane signaling receptor activity” (GO:0004888), whereas among the 23 DEGs in the cell surface receptor signaling pathway, only 3 genes had “transmembrane signaling receptor activity”. It indicates that GPCR plays a major role in the signal transduction process between cells. Therefore, the 32 DEGs with “GPCR activity” (GO:0004930) were extracted, in which 29 have both “signal transduction function” and “transmembrane signaling receptor activity”. These 29 genes were regarded as the candidate GPCR genes that act as a vital component in the embryonic reactivation of *Artemia* cyst.

### 3.4. Structure Analysis of the Candidate GPCR Genes

The structure analysis with three online software showed 11 proteins were predicted to have GPCR characteristic 7-TMHs, in which 6 proteins have obvious 7-TMHs predicted with all the three software and the other 5 proteins were confirmed by two software (Table 2, Figures S1–S3). In these proteins, evm.TU.ctg71.25 was found to have 7-THMs by TMHMM and PredictProtein, but SWISS-MODEL predicts that it has a 7 α-helix structure without transmembrane property. According to TMHMM, evm.TU.ctg310.11 is predicted to contain 7-TMHs, while the SWISS-MODEL predicts it to have two adjacent structures each with 7-TMHs. The structure of evm.TU.ctg310.11 agrees with the family C GPCRs, which have relatively large amino-terminal extracellular domains that form obligate dimers besides GPCR-defining 7-THMs domain [35].

In the 11 confirmed GPCRs, evm.TU.ctg485.29, evm.TU.ctg1078.1, evm.TU.ctg530.1, evm.TU.ctg71.25, and evm.TU.ctg179.30 were up-regulated in ArR_5h group compared to the ArD_0h group, which means they mainly function in the embryonic reactivation stage. The other six down-regulated GPCRs mainly function in the diapause stage.

### 3.5. Verification of Changes in Gene Expression for Candidate GPCRs

**Figure 8:**
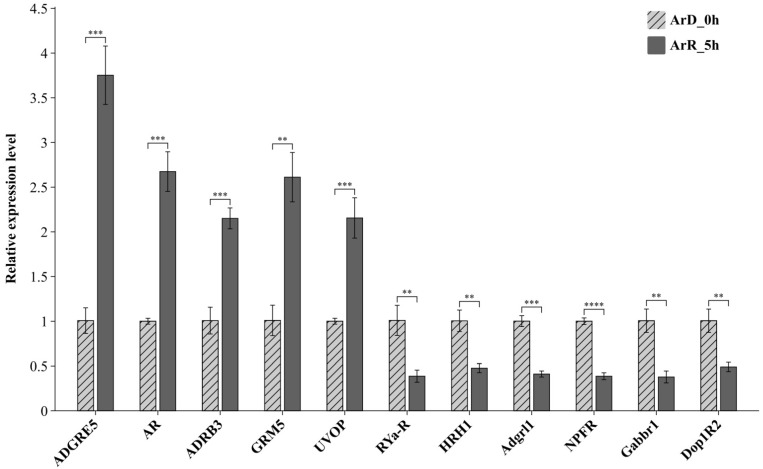
Results of RT-qPCR for the candidate genes. ‘**’ at 0.01 level. ‘***’ at 0.001 level, ‘****’ at 0.0001 level.

To verify the reliability of the GPCRs predicted above, RT-qPCR was performed to validate all the 11 genes with confirmed the GPCR characteristic structure. As shown in Figure 8, the expression of all the GPCR genes was detected in both ArD_0h and ArR_5h groups. The relative expression levels of *ADGRE5*, *AR*, *ADRB3*, *GRM5*, and *UVOP* in ArR_5h were higher than those in ArD_0h, indicating an up-regulated trend. Conversely, the relative expression levels of *RYa-R*, *CCAP-R*, *stan*, *NPFR*, *Gabbr1*, and *Dop1R2* exhibited a down-regulated trend. These findings were consistent with the results of ATAC-seq and RNA-seq.

### 3.6. Comparison with Interim Results

**Figure 9:**
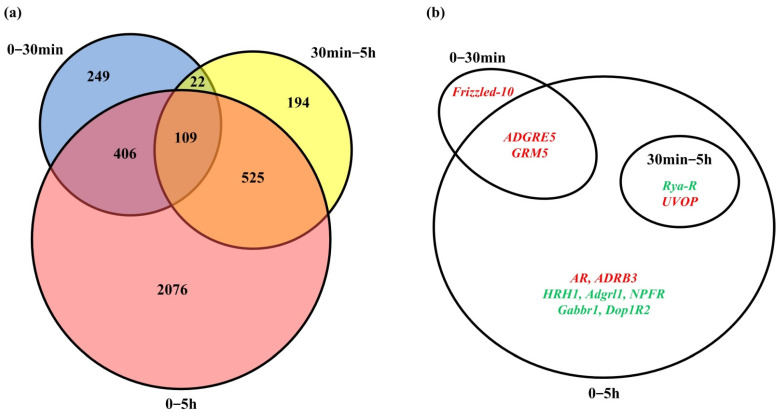
Comparison of the results among “0–30 min”, “30 min–5 h”, and “0–5 h” groups. (**a**) Comparison results of DEGs. (**b**) Comparison results of GPCR genes. The up-regulated GPCR genes were indicated in red, and the down-regulated GPCR genes were indicated in green.

As the DEGs and GPCRs of the “0–30 min” and “30 min–5 h” groups in *Artemia* cysts after diapause breaking were identified in our previously published work [36], we compared the results in this study (“0–5 h” group) with those findings. Interestingly, we found significantly more DEGs in the “0–5 h” group than the other two groups, even exceeding the combined total of the two groups (Figure 9a). There are 786 and 850 DEGs in the “0–30 min” and “30 min–5 h” groups, respectively, with 131 genes in common. The 3116 DEGs in the “0–5 h” group encompass 66% of the DEGs from the “0–30 min” group and 75% from the ‘“30 min–5 h” group, with most DEGs (2076) being unique to the “0–5 h” group. Only a very small number of genes (109) are present in all three groups. This result indicates that the reactivation of metabolism and regulation during the embryonic reactivation process might be quite gentle, and most genes do not show significant expression differences at a single stage. These active genes can only be identified through analysis over the entire time period. Therefore, even though we have analyzed the “0–30 min” and “30 min–5 h” periods, an overall analysis of gene expression changes from 0 to 5 h is still essential. Additionally, both the “0–30 min” and “30 min–5 h” groups have some unique DEGs (294 and 194 respectively), suggesting that there are variations in metabolic and regulatory processes at different stages of embryonic reactivation.

The signaling pathways involved in the three groups are essentially the same, with GPCR and cell-surface receptor signaling pathways being the primary ones. The Wnt, Notch, and enzyme-linked receptor protein signaling pathways are active throughout the entire 5 h after diapause breaking. Among the identified GPCR genes, both the two GPCR genes from the “30 min–5 h” group also present in the “0–5 h” group. Of the three GPCR genes from the “0–30 min” group, two are present in the “0–5 h” group, while the *Frizzled-10* gene is only active within the first half hour after diapause breaking. Additionally, seven GPCR genes were identified exclusively in the “0–5 h” group, which may be corresponding to the gentle metabolic and regulatory changes during the embryonic reactivation process (Figure 9b).

## 4. Discussion

### 4.1. Signal Regulation of Embryonic Reactivation Process

Embryonic diapause, as a key survival strategy evolved over time, can temporarily suspend embryonic development before seasonal adversity arrives, causing it to stagnate at a specific developmental stage, waiting for appropriate growth environment signals to stimulate the breaking of dormancy again and continue to develop into mature embryos [37]. This phenomenon has been frequently exploited across the animal kingdom [38]. The widespread distribution also suggests that this phenomenon is very ancient in evolution and may have a unified molecular basis. Diapause may occur at various developmental stages due to different species, typically accompanied by decreased metabolic levels, cell cycle arrest, enzyme activity inhibition, and modification and degradation regulation of biological macromolecules [39]. The occurrence, maintenance, and breaking of diapause are all controlled by complex gene regulatory networks that need to be explored, and are closely related to the changes in environment, endocrine, rhythm, self transcriptome, and proteome levels. *Artemia*, as a representative dormant species of the crustacean arthropod phylum, is one of the classic animal models for diapause research. For the purpose of better understanding the embryonic reactivation process after diapause breaking, in this work, the *Artemia* cysts in diapause (ArD_0h) and embryonic reactivation stages (ArR_5h) were selected for high-throughput sequencing. The samples for embryonic reactivation stage was extracted from the cysts at 5 h after diapause breaking, which is the period with most active metabolism after diapause breaking [26]. In order to improve the accuracy of high-throughput data, ATAC-seq and RNA-seq data were combined to confirm each other and obtain more reliable results. The integrated analysis showed that during the embryonic reactivation process, the metabolism and regulation of *Artemia* cysts were significantly changed but the change is gentle based on the comparison result to the “0–30 min” and “30 min–5 h” groups. Both ATAC-seq and RNA-seq analysis results show that more genes are up-regulated in the ArR_5h group. After diapause breaking, the metabolic process in *Artemia* embryo is restarted. The metabolic and regulatory processes are much more active than those in the diapause state. Therefore, it is not surprising that more genes are up-regulated. In the regulation process of embryonic reactivation, signal transduction regulation plays a very important role. The current research on the molecular regulatory mechanism of *Artemia* embryonic reactivation is still poor. Through the GO and KEGG enrichment analysis on the integrated analysis, it was found that there are multi-signal transduction pathways involved in regulation of embryonic reactivation. The GPCR signaling pathway, cell surface receptor signaling pathway, hormone-mediated signaling pathway, Wnt, Notch, mTOR, AGE-RAGE, FoxO, neuropeptide, enzyme linked receptor protein signaling pathways, and Phosphatidylinositol signal transduction systems were all involved. It indicates that embryonic reactivation regulation is a complex regulatory process mediated by multiple signaling systems. The Wnt signaling pathway, known for its high conservation and complexity, is one of the most crucial developmental pathways that govern the cell fate decisions and tissue patterning. Jia et al. have found that Wnt signaling pathway is related to *Artemia* embryonic reactivation process [27]. TOR signaling pathway is highly conserved for its role in integrating signals related to many biological process such as nutrient and energy availability, cell growth, development, and metabolism. Many studies have also shown that mTOR regulates the diapause in mammals and insects although its mechanism is still poorly understood [40,41]. The Notch signaling pathway affects multiple processes involved in the normal morphological development of cells, including differentiation of progenitor cells, apoptosis, proliferation, and formation of cell boundaries [42]. Ouellet et al. proved that the Notch signaling pathway plays a role in both maintaining and terminating diapause in *Caenorhabditis. elegans* embryos [43].

### 4.2. Other Pathways Involved in Embryonic Reactivation

Based on the KEGG pathway enrichment analysis of the integrated DEGs, besides signaling pathways, there are also many integrated DEGs distributed in other pathways such as endocytosis, carbon metabolism, and autophagy—animal pathways. Wang et al. [41] have found that the diapause development of *Bactrocera minax*, is closely associated with endocytosis, core metabolic pathways of carbohydrates, and autophagy—animal pathways. They presumed that the activation of endocytosis was a concomitant of restored metabolism and augmented signaling. Autophagy is a vital and evolutionarily conserved process that involves the degradation of cytoplasmic components, including macromolecules and organelles, within lysosomes or vacuoles. This process facilitates their recycling in response to various physiological activities [44]. Autophagy signaling pathway has been demonstrated to play roles in the diapause development of *Artemia parthenogenetica* [45]. In addition, autophagy has been found to be regulated by the TOR signaling pathway [46]. Therefore, it is not surprising that the mTOR and autophage signaling pathways are both found closely related to the embryonic reactivation in this work.

### 4.3. GPCRs Participating in Artemia Embryonic Reactivation Process

GPCRs are widely existing signal receptors that have been found in all organisms from yeast to humans and play an important role in various biological signal transduction processes. Multiple GPCR genes typically exist in a species, for example, there are 800 GPCR genes in human [47] and 1783 GPCR genes found in mouse genomes [48]. In the *Takifugu rubripes*, 298 GPCR genes were detected that were grouped into five families [49]. GPCRs, with diapause hormones acting as ligands in insects, suggest that GPCRs could be involved in the signal transduction processes of diapause [50,51]. In this work, we recognized 32 genes exhibiting GPCR activity (GO: 0004930 G-protein coupled receptor activity) in *Artemia* cysts through GO enrichment analysis. Then, 29 possible GPCR genes were screened with signal transduction function and transmembrane signaling receptor activity. According to the unique 7-THMs structure of GPCR, 11 genes were determined to encode the proteins structurally consistent with GPCR characteristics. Among them, five genes were up-regulated, which mainly function in 5 h after cysts reactivation, while six genes were down-regulated, which may mainly play a role in the diapause cysts. The gene expression of these candidate GPCRs were all further validated by RT-qPCR experiments.

## 5. Conclusions

Embryonic diapause is a common evolutionary adaptation observed across a wide range of organisms. The current research on embryonic diapause mostly focuses on the initiation and maintenance of diapause, but there is little research on the mechanism of embryonic reactivation phenomenon. In this work, two high-throughput sequencing methods, ATAC-seq and RNA-seq, were integrated to study the signal regulation process of embryonic reactivation in *Artemia*. The results showed that after 5 h of diapause breaking, the metabolism and regulation of *Artemia* cysts were quite active, involving multiple signal transduction pathways such as GPCR, Wnt, Notch, mTOR etc. It indicates that embryonic reactivation is a complex process regulated by multiple signal pathways. Through the GO and KEGG enrichment analysis of the high-throughput data, combined with the protein structure analysis and RT-qPCR validation, 11 genes were identified that conform to GPCR characteristics. Among them, five genes may participate in the embryonic reactivation process, while the other six genes may contribute to the signal transduction in diapause cyst. These results provide important clues for the in-depth research on the regulation mechanisms of diapause and embryonic reactivation process in *Artemia* cyst.

## Figures and Tables

**Table 1:** Comparison of DEGs from ATAC-seq and RNA-seq.

Subject	Up-Regulated	Down-Regulated	Total
ATAC-seq	7124	5399	9159
RNA-seq	3608	3251	6859
Integrated DEGs	2005	1111	3116

**Table 2:** Analysis of the structures for the candidate GPCR proteins.

Candidate GPCR Genes	Gene Name	TMHMM	PredictProtein	SWISS-MODEL
evm.TU.ctg485.29	*ADGRE5*	7	7	7
evm.TU.ctg1078.1	*AR*	7	7	7
evm.TU.ctg530.1	*ADRB3*	7	7	7
evm.TU.ctg71.25	*GRM5*	7	7	7
evm.TU.ctg179.30	*UVOP*	6	7	7
evm.TU.ctg441.12	*RYa-R*	7	7	7
evm.TU.ctg428.23	*HRH1*	7	7	7
evm.TU.ctg201.8	*Adgrl1*	7	7	7
evm.TU.ctg54.13	*NPFR*	7	7	6
evm.TU.ctg310.11	*Gabbr1*	7	6	7 × 2
evm.TU.ctg394.6	*Dop1R2*	6	7	7
evm.TU.ctg3.36	*gar-2*	6	6	5
evm.TU.ctg92.26	*CCAP-R*	5	6	5
evm.TU.ctg150.6	*stan*	5	6	0
evm.TU.ctg288.3	*Cirl*	5	5	5
evm.TU.ctg73.14	*QRFPR*	5	5	6
evm.TU.ctg640.11	*SCOP1*	4	5	5
evm.TU.ctg195.9	*CCKAR*	4	4	5
evm.TU.ctg275.30	*TkR86C*	4	4	4
evm.TU.ctg889.7	*moody*	4	4	2
evm.TU.ctg116.37	*mth2*	3	3	4
evm.TU.ctg155.21	*HRH1*	3	3	3
evm.TU.ctg16.15	*7tm_1*	3	3	3
evm.TU.ctg397.21	*Svep1*	2	1	0
evm.TU.ctg88.22	*mAChR-A*	1	1	1
evm.TU.ctg469.18	NA	1	1	0
evm.TU.ctg25.48	*Pep-like*	0	0	0
evm.TU.ctg212.15	*SCAF8*	0	0	0
evm.TU.ctg115.32	*FKBP4*	0	0	0

## Data Availability

Data are contained within the article and Supplementary Files. The original contributions presented in the study are included in the article and Supplementary Files, further inquiries can be directed to the corresponding author.

## Supplementary Materials

The following supporting information can be downloaded at: https://www.mdpi.com/article/10.3390/genes15081083/s1, Figure S1: Prediction results of TMHMM, Figure S2: Prediction results of PredictProtein, Figure S3: Prediction results of SWISS-MODEL; Table S1: Primer sequences used for RT-qPCR, Table S2: The GO, KEGG annotations, and Padj values of integrated DEGs based on the ATAC-seq and RNA-seq datasets, Table S3: 3 Prediction results of TMHMM.

## Abbreviations

TMH	Trans-membrane helix
DEGs	Differentially expressed genes
GPCR	G-protein-coupled receptor
TSS	Downstream gene start site
