# Supplementary figures and images for: Integrating ATAC-Seq and RNA-Seq Reveals the Signal Regulation Involved in the Artemia Embryonic Reactivation Process
Source: Genes (Basel). 2024 Aug 16;15(8):1083. doi: 10.3390/genes15081083 (PMC11353689; doi:10.3390/genes15081083)

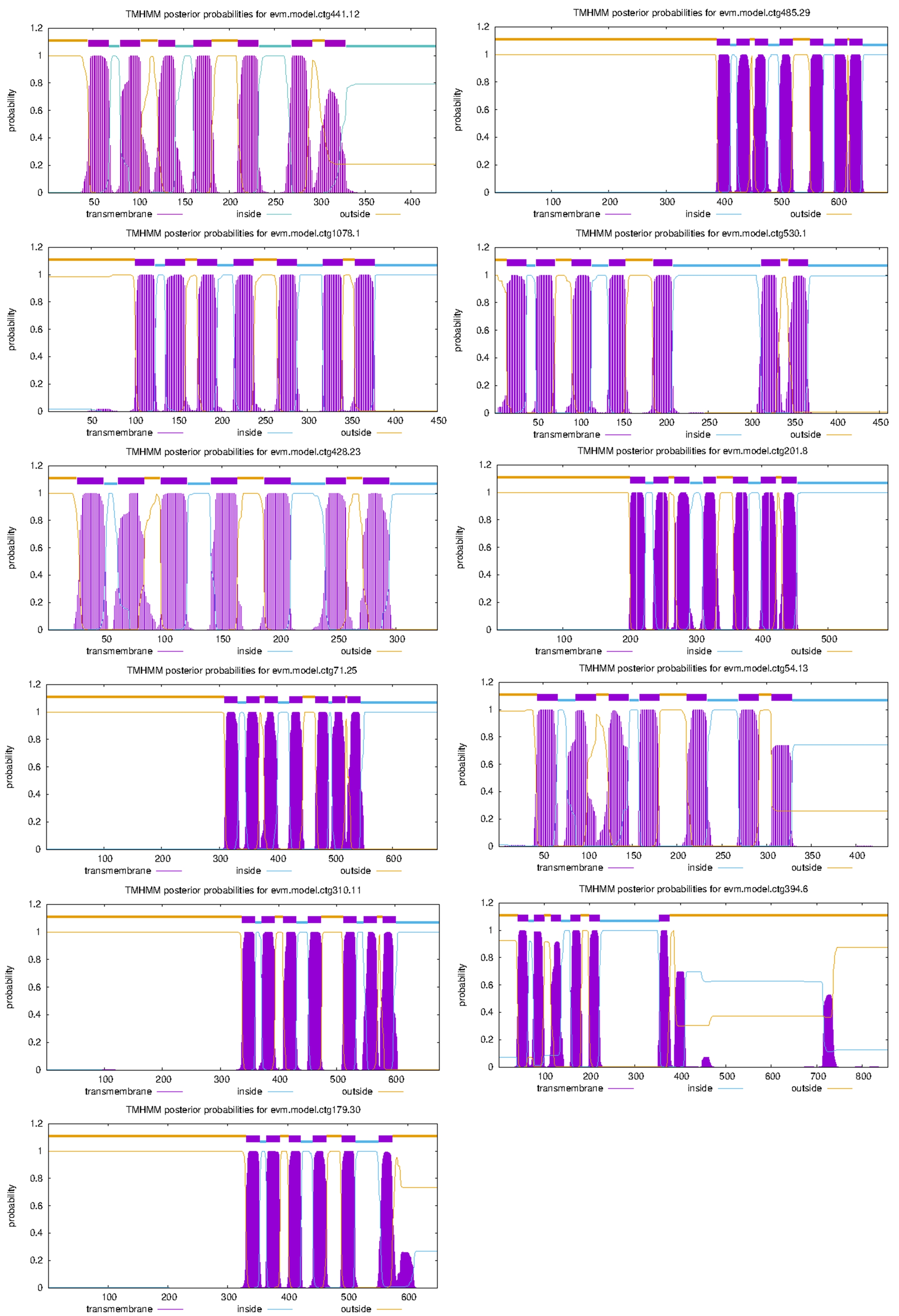

Supplement: Supplementary file 1 [file genes-15-01083-s001.zip › Figure S1.jpg]

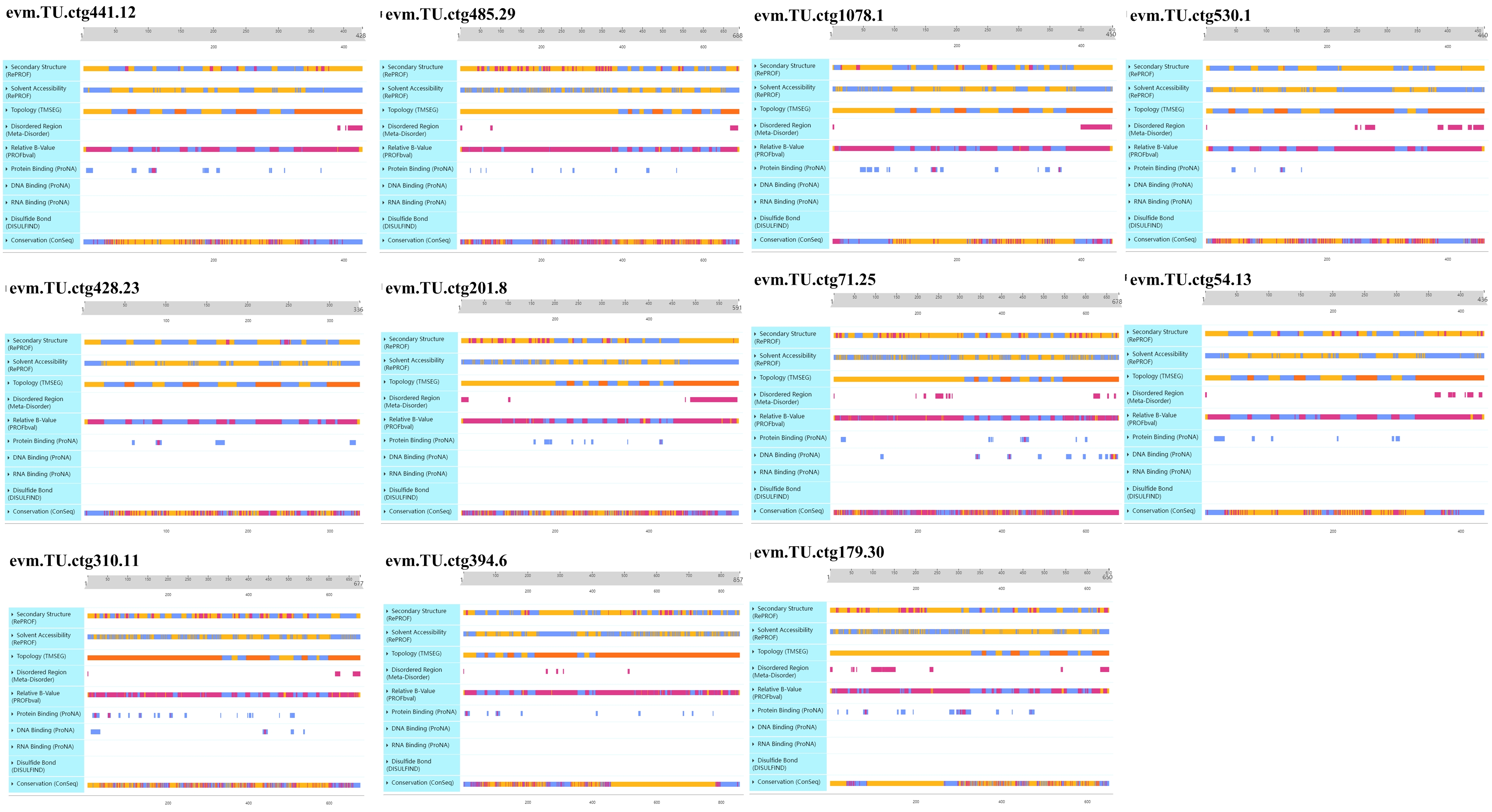

Supplement: Supplementary file 1 [file genes-15-01083-s001.zip › Figure S2.jpg]

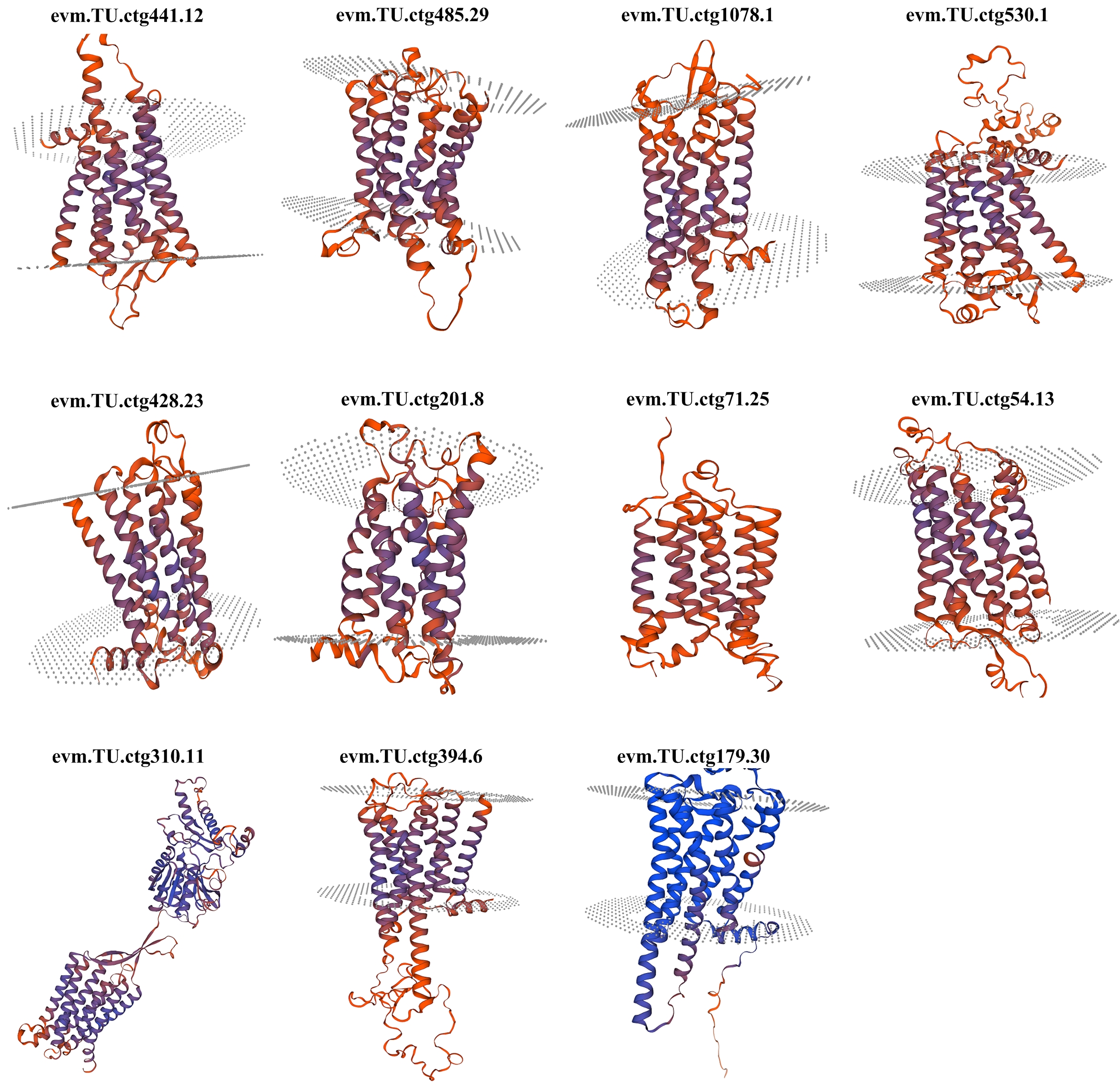

Supplement: Supplementary file 1 [file genes-15-01083-s001.zip › Figure S3.jpg]
